# Transcriptional Activity and Stability of CD39+CD103+CD8+ T Cells in Human High-Grade Endometrial Cancer

**DOI:** 10.3390/ijms21113770

**Published:** 2020-05-27

**Authors:** Hagma H. Workel, Nienke van Rooij, Annechien Plat, Diana C.J. Spierings, Rudolf S. N. Fehrmann, Hans W. Nijman, Marco de Bruyn

**Affiliations:** 1 Department of Obstetrics and Gynaecology, University Medical Center Groningen, University of Groningen, 9713 GZ Groningen, The Netherlands; h.h.workel@umcg.nl (H.H.W.); n.van.rooij@umcg.nl (N.v.R.); a.plat@umcg.nl (A.P.); h.w.nijman@umcg.nl (H.W.N.); 2 European Research Institute for the Biology of Ageing, University Medical Center Groningen, University of Groningen, 9713 GZ Groningen, The Netherlands; d.c.j.spierings@umcg.nl; 3 Department of Medical Oncology, University Medical Center Groningen, University of Groningen, 9713 GZ Groningen, The Netherlands; r.s.n.fehrmann@umcg.nl

**Keywords:** tissue-resident memory cell, endometrial cancer, CD103, CD39, transcript stability, cytokinesis, IL-21

## Abstract

Tumor-infiltrating CD8+ T cells (TIL) are of the utmost importance in anti-tumor immunity. CD103 defines tumor-resident memory T cells (T_RM_ cells) associated with improved survival and response to immune checkpoint blockade (ICB) across human tumors. Co-expression of CD39 and CD103 marks tumor-specific T_RM_ with enhanced cytolytic potential, suggesting that CD39+CD103+ T_RM_ could be a suitable biomarker for immunotherapy. However, little is known about the transcriptional activity of T_RM_ cells in situ. We analyzed CD39+CD103+ T_RM_ cells sorted from human high-grade endometrial cancers (*n* = 3) using mRNA sequencing. Cells remained untreated or were incubated with PMA/ionomycin (activation), actinomycin D (a platinum-like chemotherapeutic that inhibits transcription), or a combination of the two. Resting CD39+CD103+ T_RM_ cells were transcriptionally active and expressed a characteristic T_RM_ signature. Activated CD39+CD103+ T_RM_ cells differentially expressed *PLEK*, *TWNK*, and *FOS*, and cytokine genes *IFNG*, *TNF*, *IL2*, *CSF2* (GM-CSF), and *IL21*. Findings were confirmed using qPCR and cytokine production was validated by flow cytometry of cytotoxic TIL. We studied transcript stability and found that PMA-responsive genes and mitochondrial genes were particularly stable. In conclusion, CD39+CD103+ T_RM_ cells are transcriptionally active T_RM_ cells with a polyfunctional, reactivation-responsive repertoire. Secondly, we hypothesize that differential regulation of transcript stability potentiates rapid responses upon T_RM_ reactivation in tumors.

## 1. Introduction

The influence of tumor-infiltrating lymphocytes (TIL) on cancer prognosis is widely recognized, and TIL are studied in a wide variety of solid tumors. The composition of the immune infiltrate is of the utmost importance, as the immune architecture mainly determines whether the balance tips towards an anti-tumor or pro-tumor immune response [1]. CD103, the αE subunit of integrin αEβ7, defines intra-epithelial resident memory T cells (T_RM_ cells) with increased cytolytic potential, improved immune synapse formation, and increased tumor antigen sensitivity [2,3,4]. CD8+ T cells upregulate CD103 upon combined TCR stimulation and TGF-β signaling [5,6,7]. Even though TGF-β production is commonly attributed to dendritic cells and T regulatory cells, differentiated CD103+ T_RM_ are also capable of self-producing activated TGF-β1 to maintain CD103 expression on their cell surface [8]. CD103+ T_RM_ are associated with prolonged survival in many solid tumors [4,6,9,10,11,12,13], including endometrial cancer (EC). Endometrial tumors are stratified into risk groups based on histology, grade, and FIGO stage, among others [14]. In addition, EC can be classified according to molecular subtype: copy number low/p53 wild-type, copy number high/p53 mutant, polymerase E mutant (POLE-mutant), and mismatch repair deficient (dMMR/MSI). POLE-mutant, dMMR, and p53-aberrant tumors are typically high-grade tumors [15]. Particularly, POLE-mutant and dMMR tumors harbor many neoantigens and are more infiltrated by immune cells, including CD103+ T_RM_ [16,17,18]. CD103+ T_RM_, both in tumor and non-tumor tissue, are marked by expression of *PDCD1*, *ITGAE*, *CXCR6*, and *SPRY1* in lung cancer [19]. Tumor-resident CD103+ T_RM_ expressed a unique genotype compared to non-tumor CD103+ T_RM_, characterized by expression of *ENTPD1* (CD39) [20]. Indeed, bystander, i.e., non-tumor specific T cells, lack CD39 expression [21]. CD39, also known as ectonucleoside triphosphate diphosphohydrolase 1 (ENTPD1), catalyzes the phosphohydrolysis of extracellular ATP and ADP to eventually synthesize immunosuppressive adenosine. CD39 is upregulated on activated T cells [22], regulates T cell activation and polarization, and is considered an immunosuppressive marker associated with T cell exhaustion [23,24]. CD39 has therefore been put forth as an immunoregulatory checkpoint and a new therapeutic target in cancer [25]. A further specification of T_RM_ cells in endometrial cancer might therefore be relevant, as CD39 and CD103 co-expression identifies tumor-resident, clonally expanded, tumor antigen-specific T cells with superior cytolytic capacity [19,20]. Moreover, tumor-resident CD103+ T_RM_ differentially express immune checkpoints such as *CTLA4, TIM3, LAG3*, and *TIGIT*, indicating T cell exhaustion due to excess antigen stimulation [9,18,19,26]. In line with this, CD103+ T_RM_ are linked to response to immune checkpoint blockade (ICB) [27,28]. Thus, it can be hypothesized that patients with both a sufficient number of T_RM_ cells and a specific subtype of T_RM_ cells are likely to respond to immunotherapy/immune checkpoint blockade. However, little is known about transcriptional activity of T_RM_ cells in situ. We studied the transcriptional profile of high-grade endometrial cancer CD39+CD103+ T_RM_ cells in situ, after T cell activation, and after transcriptional inhibition with actinomycin D in order to elucidate core elements necessary for successful T cell reactivation. Moreover, we studied the immune profile of TIL in the context of pretreatment with actinomycin D, a platinum-like chemotherapeutic. In this study we showed that resting CD39+CD103+ T_RM_ cells were transcriptionally active and expressed a characteristic tissue-resident transcriptional profile. Upon activation, T_RM_ cells upregulated markers of T cell activation, cytolytic activity, and cytokine production. Secondly, we studied transcript stability and found that PMA-responsive immune genes and mitochondrial genes were particularly stable, which led us to hypothesize this differential regulation of transcript stability potentiates rapid responses upon T_RM_ reactivation in tumors.

## 2. Results

### 2.1. CD39+CD103+ T_RM_ and CD8+ TIL are Transcriptionally Active in Situ

**Figure 1:**
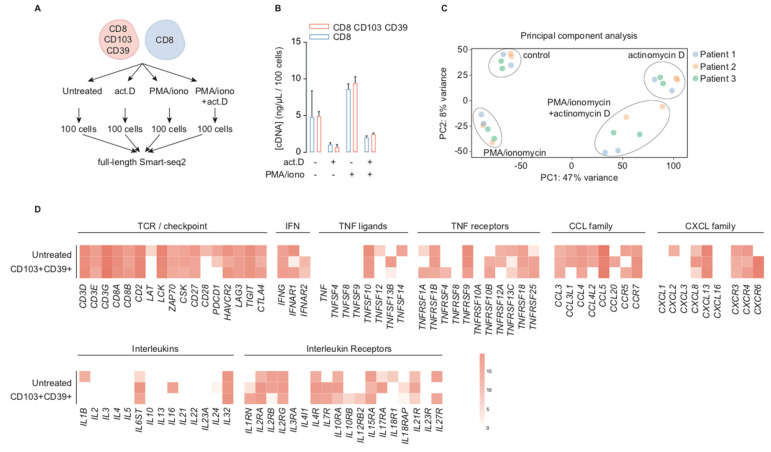
CD39+CD103+ T_RM_ cells sorted from human endometrial tumors are transcriptionally active. (**A**) Overview of experimental set-up. CD39+CD103+ T_RM_ cells and CD8+ TIL were sorted from human high-grade endometrial cancer digests (*n* = 3). Subsequently, cells remained untreated or were treated for 4.5 h with actinomycin D, or 4 h of PMA/ionomyin or pre-incubation for 30 min with actinomycin D followed by 4 h PMA/ionomycin. (**B**) Concentration of cDNA (ng/μL/100 cells) of CD39+CD103+ T_RM_ cells and CD8+ TIL per treatment group. The median concentration + 95% confidence interval is depicted. (**C**) Principal component analysis of mRNA sequencing data of all T_RM_ samples. The first two principal components are depicted. Individual patient samples are identified by separate colors. (**D**) Heatmap of a customized set of T cell markers of library size-normalized, log2-transformed counts of untreated CD39+CD103+ T_RM_ cells from three patients. The scale applies to all similar heatmaps of CD39+CD103+ T_RM_ cells throughout the paper.

We analyzed the transcriptome of CD39+CD103+ T_RM_ and a reference population of CD8+ TIL in high-grade endometrial cancer using mRNA sequencing (summarized in Figure 1A). The reference population was randomly sorted from the CD3+CD8+ population of each of the tumor digests, but for further information, the CD39/CD103 staining of these tumors were added (Supplementary Figure S1A).

To compare transcriptional profiles in resting condition to the transcriptional profile upon activation and transcriptional inhibition, samples were either not treated (untreated) or treated with PMA/ionomycin (PMA), actinomycin D (ACT), or a combination of both. Actinomycin D is a chemotherapeutic with some similarities to platinum-based chemotherapy and is therefore also of interest in immune cells in endometrial cancer. cDNA concentrations were comparable between CD39+CD103+ T_RM_ and CD8+ bulk cells (Figure 1B). However, there were distinct differences between treatment conditions: the cDNA concentration increased upon activation with PMA/ionomycin and diminished upon transcriptional inhibition by actinomycin D independently of PMA treatment (T_RM_ median 4.92 ng/μL/100 cells untreated, 0.70 ng/μL/100 cells actinomycin D, 9.44 ng/μL/100 cells PMA, and 2.48 ng/μL/100 cells combination, respectively). Principal component analysis identified actinomycin D treatment as the first component, explaining 47% of variance, and PMA/ionomycin as the second with a variance of 8% (Figure 1C). Resting, untreated, CD39+CD103+ T_RM_ cells robustly transcribed characteristic CD8+ T cell markers such as *CD3D*, *CD3E*, *CD3G*, *CD8A*, *CD8B* and *CD27* (Figure 1D). In addition, they expressed several checkpoints frequently found in T_RM_ such as *CTLA4*, *TIGIT*, *LAG3*, *HAVCR2*, and *PDCD1*. Expression of several other T cell-related genes in resting CD39+CD103+ T_RM_ varied: *IFNG* was uniformly expressed, but several TNF ligands, TNF receptors, chemokines, and chemokine receptors were expressed in some, but not all patient samples. It was noteworthy that *TNF*, *TNFSF4*, *TNFSF8*, and *TNFSF9*, all associated with T cell activation, were barely transcribed at baseline, as were most cytokine-related genes. We observed a similar expression pattern in CD8+ bulk TIL (Supplementary Figure S1D). These findings indicate that transcription in CD39+CD103+ T_RM_ cells is ongoing during in vitro culture, and these T cells have baseline transcript expression despite their exhausted phenotype.

### 2.2. Transcription Inhibition by Actinomycin D Reveals Distinct Differences in Transcript Stability

**Figure 2:**
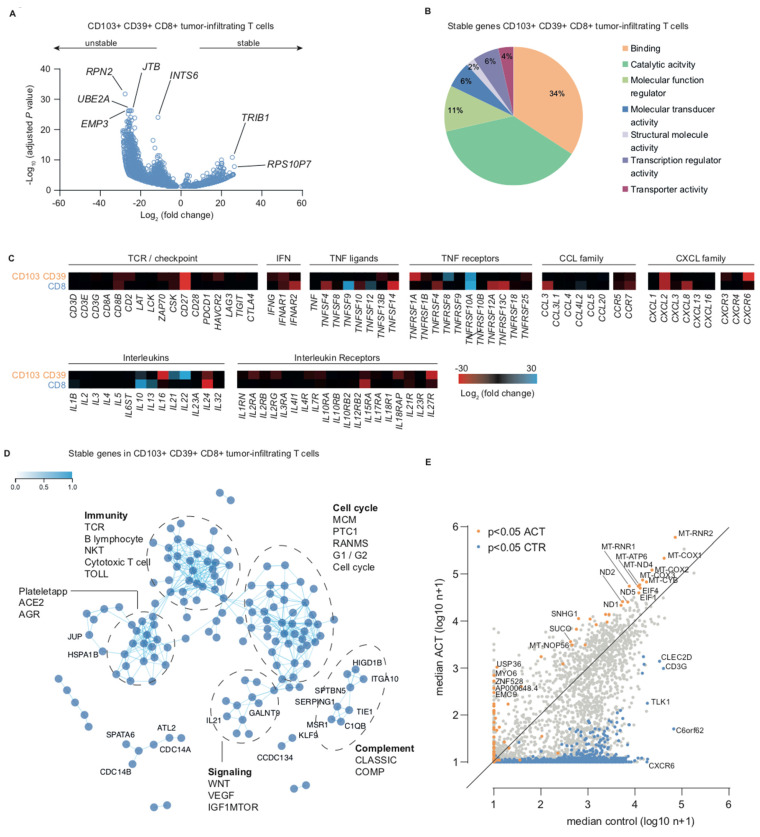
Effect of actinomycin D on transcriptional activity of CD39+CD103+ T_RM_. (**A**) Volcano plot depicting differentially expressed genes (DEseq2) of CD39+CD103+ T_RM_ cells treated with actinomycin D for 4.5 h versus untreated. Each dot represents a gene with a Benjamini–Hochberg adjusted *p*-value < 0.05. Differentially decreased genes are depicted as unstable, differentially increased genes as stable. (**B**) Gene ontology analysis of top-250 differentially stable genes performed by Panther DB. (**C**) Fold changes of a customized set of T cell markers upon actinomycin D treatment versus untreated CD39+CD103+ T_RM_ and CD8+ TIL. (**D**) Co-functionality network of the top-250 differentially expressed genes in actinomycin D versus untreated CD39+CD103+ T_RM_ cells with default parameters. Several Biocarta pathways with z-score >2 are annotated. (**E**) Median gene expression values of CD39+CD103+ T_RM_ cells (log10 n+1), with the median value of untreated cells on the *x*-axis and of actinomycin D samples on the *y*-axis. Significant differentially expressed genes (DESeq2) are depicted for untreated (blue) and actinomycin D (orange) cells.

We studied transcript turnover and transcript stability in CD39+CD103+ T_RM_ cells and CD8+ TIL by comparing untreated and transcriptionally halted samples treated with actinomycin D. We observed a strong decrease in the number of transcripts in both CD39+CD103+ T_RM_ and CD8+ TIL. This was also reflected in the distribution of the volcano plots, with a distinctly larger negative fold change-tail (Figure 2A, Supplementary Figure S1B, Supplementary Table S1, and Supplementary Table S2).

In actinomycin D-treated samples, 3839 genes were differentially lower expressed compared to untreated T_RM_. Top differentially expressed unstable genes in actinomycin D-treated versus untreated cells included *RPN2* (ribophorin II, endoplasmatic reticulum gene involved in Golgi transport), *INTS6* (integrator complex subunit 6, a putative RNA helicase that interacts with RNA polymerase II), *EMP3* (epithelial membrane protein 3, involved in cell proliferation and cell–cell interactions), *JTB* (jumping translocation breakpoint, a requirement for cytokinesis during mitosis), and *UBE2A* (ubiquitin conjugating enzyme E2 A, an enzyme required for post-replicative DNA damage repair). Top differentially expressed stable genes in actinomycin D-treated versus untreated samples included *TRIB1* (tribbles pseudokinase 1, encoding an adapter protein involved in protein degradation) and *RPS10P7* (ribosomal protein S10 pseudogene 7) (Figure 2A). In bulk CD8+ TILs, *SDHB*, *MCAT*, and *CD27* were the top differentially expressed unstable genes, and *IBA57* was one of the top stable genes (Supplementary Figure S1B). Notably, many unstable CD8+ TIL genes were also differentially unstable in CD39+CD103+ T_RM_ (Supplementary Table S1 and Supplementary Table S2). To determine the molecular function of stable transcripts, we classified the top-250 differentially expressed stable genes in CD39+CD103+ T_RM_ according to the Gene Ontology (GO) molecular function. 71% of stable genes were appointed to GO functions binding or catalytic activity (Figure 2B and Supplementary Table S3). Actinomycin D is a chemotherapeutic that inhibits transcription, but it is sometimes also used as a read-out for platinum-based chemotherapy. We therefore further elaborated on the effect of actinomycin D on cytotoxic T cell characteristics and hallmark functions by comparing fold-changes of key immune-related genes. Expression of several TNF ligands and receptors (*TNFSF4*, *TNFSF14*, *TNFRSF1A*, *TNFRSF4*, *TNFRSF12A*, and *TNFRSF13C*) showed a negative fold change upon actinomycin D treatment, as did cytokine genes *CCL3*, *CXCL2*, *CXCL8*, and *CXCR6* and interleukin (receptor) genes *IL16*, *IL24*, *IL15RA*, and *IL27R* (Figure 2C). By contrast, *TNFSF9*, *TNFRSF10A*, *IL10*, *IL13*, *IL21*, and *IL22* were relatively stable under actinomycin D. To study the pathway involvement of stable genes in CD39+CD103+ T_RM_, we generated a co-functionality network with an integrative tool that predicts gene functions based on a guilt-by-association (GBA) strategy utilizing >106,000 expression profiles [29]. The top-250 stable genes (maximum number for this analysis) were analyzed with default parameters for Biocarta pathways (Figure 2D). We observed two large sub-networks: genes in sub-network 1 were associated with or assigned to take part in the cell cycle, whereas sub-network 2 contained immunity-related genes. Two smaller sub-networks were associated with complement and Wnt/VEGF/mTOR signaling (Figure 2D). Interestingly, among the top pathways in the immune cluster were not only the T cell receptor pathway and cytotoxic T cell/cytotoxicity pathway, but also the B lymphocyte and NK-T pathways. We have previously hypothesized that CD103+ TIL affect B cell migration via CXCL13 production, which may in part explain the association with B cell-related genes/pathways [18].

Although most transcripts decreased after actinomycin D treatment, we also noticed a steady or even increased number of transcripts for some genes. Thus, we compared median counts of untreated samples and actinomycin-treated samples (Figure 2E). Many of the significantly enriched transcripts were mitochondrial genes, such as *MT-ND4*, *MT-CYB*, *MT-COX1*, *MT-COX2*, and *MT-ATP6*. Median expression of *EIF1* and *EIF4*, both associated with translation initiation, also increased upon actinomycin treatment. Several genes were not expressed in untreated samples but were found in actinomycin D-treated cells, suggesting de novo synthesis due to transcriptional arrest. These genes included *USP36* (deubiquitinase), *MYO6* (actin-based motor molecules), *ZNF528* (DNA-binding transcription activity), *AP000648.4* and *EMC9* (ER membrane protein). Taken together, we found increased transcript stability in cell cycle and immunity-related genes, in particular in several T_RM_-associated genes and mitochondrial genes.

### 2.3. Stimulation with PMA/Ionomycin Induces an Activation-Responsive Genotype in Tumor-Infiltrating Lymphocytes

**Figure 3:**
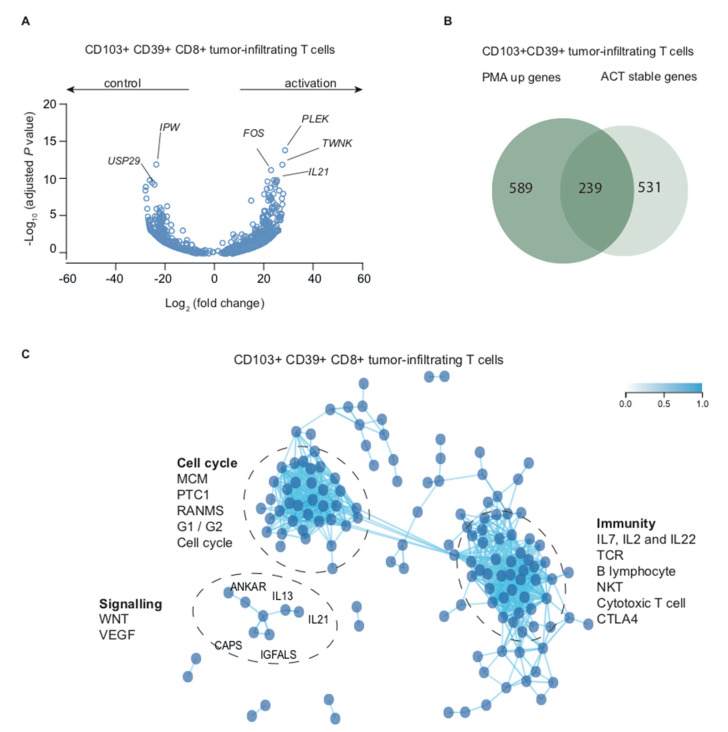
CD39+CD103+ T_RM_ cells differentially upregulate genes associated with immunity, cell cycle, and the tumor microenvironment. (**A**) Volcano plot depicting differentially expressed genes (DEseq2) of CD39+CD103+ T_RM_ cells treated with PMA/ionomycin for four hours versus untreated cells. Each dot represents a gene with a Benjamini–Hochberg adjusted *p*-value < 0.05. (**B**) Venn diagram showing overlap of differentially expressed genes of PMA/ionomycin-treated CD39+CD103+ T_RM_ cells and significantly stable genes after actinomycin D treatment. (**C**) Co-functionality network of the top-250 differentially expressed genes in PMA/ionomycin-treated cells versus untreated CD39+CD103+ T_RM_ cells with default parameters. Several Biocarta pathways with z-score > 2 are annotated.

To elucidate biological and transcriptional processes in human tumor-associated T_RM_ cells upon re-activation, we analyzed CD39+CD103+ T_RM_ cells and heterogeneous CD8+ TIL treated with PMA/ionomycin. Activated CD39+CD103+ T_RM_ differentially expressed 589 genes (Supplementary Table S4). *TWNK* (twinkle mtDNA helicase, involved in mitochondrial DNA replication and energy formation), *PLEK* (pleckstrin, induces reorganization of the cytoskeleton, involved in lymphocyte spreading and immune synapse formation), *IL21* (interleukin 21) and *FOS* (FOS proto-oncogene) were top hits in this analysis (Figure 3A).

*TWNK*, *PLEK*, *IL21*, and several FOS gene family members (*FOSL*, *FOSB*) were also differentially expressed in CD8+ TIL (Supplementary Figure S1C and Supplementary Table S5), but *FOS* was not. *FOS* is thought to play an important role in signal transduction, cell proliferation, and differentiation via SMAD3/SMAD4/JUN/FOS complex upon TGF-β stimulation. Indeed, it is very likely that CD103+, but not CD8+ bulk TIL were exposed to TGF-β in vivo, as we and others have previously shown that CD103 expression is TGF-β dependent [5,6]. Other differentially expressed genes in activated T_RM_ included *SPRY1*, *SPRY2*, *NR4A2*, and *NR4A3* (Supplementary Table S3), which are all negative feedback mechanisms to limit T cell activation. Activated CD8+ TIL top differentially expressed genes were *IL21*, *RCL1* (RNA terminal phosphate cyclase like 1, involved in rRNA processing), and *EGR2* (early growth response 2, transcription factor with three tandem C2H2-type zinc fingers) (Supplementary Figure S1C). Several cytokines were differentially expressed in both CD39+CD103+ T_RM_ and CD8+ bulk including *IL2*, *IL10*, *IL13*, *IL17A*, *IL21*, and *IL22*, as were chemokines *CCL3*, *CCL4*, and *CXCL3* and markers of T cell activation *LTA*, *TNF*, *TNFSF9*, and *TNFRSF10A* (Figure 3A, Supplementary Figure S1D, Supplementary Table S4, and Supplementary Table S5). Interestingly, several genes that were relatively stable upon actinomycin treatment were particularly responsive to PMA/ionomycin (Figure 3B and Supplementary Figure S1E).

To obtain an idea about the pathways involved in PMA-responsive genes, we entered the top-250 differentially increased genes in PMA-treated CD39+CD103+ T_RM_ in the co-functionality network analysis as described previously. Within this cohort, we observed two large sub-networks of genes, one representing several cell cycle pathways and one highly involved in adaptive immunity (Figure 3C). The overlap in sub-network formation between actinomycin-stable genes and PMA-upregulated genes may in part be explained by the observed link between genes in these groups (Figure 3B). A third smaller, but distinctly separate, network of *IL13*, *IL21*, *ANKAR*, *CAPS*, and *IGFALS* was associated with Wnt and VEGF signaling, and are likely associated with environmental cues in high-grade endometrial cancer. Activated CD39+CD103+ T_RM_ cells thus expressed several classical and non-classical CD8 T cell cytokines, various markers of T cell activation, and genes associated with cell cycle and adaptive immunity.

### 2.4. Cytotoxic T Cells in Endometrial Cancer are Polyfunctional T Cells that can Produce IL-21 and GM-CSF

**Figure 4:**
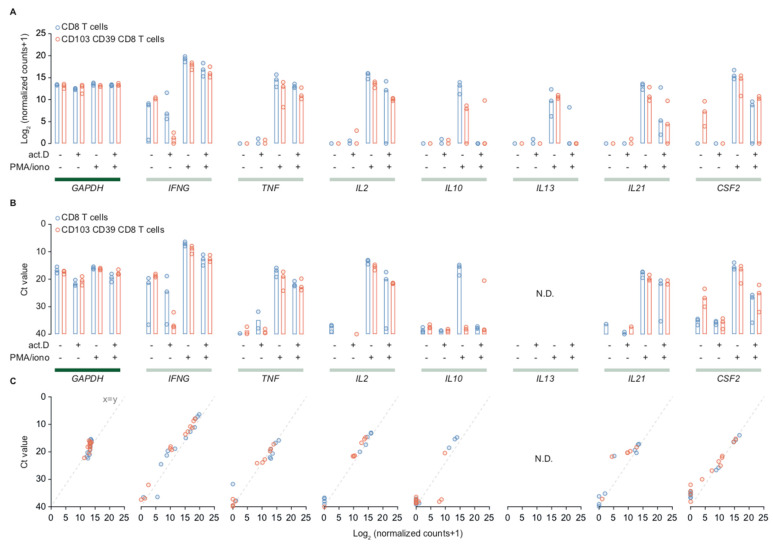
CD39+CD103+ T_RM_ and CD8+ TIL robustly express cytokine genes upon stimulation with PMA/ionomycin. (**A**) Overview of log2-normalized counts of CD39+CD103+ T_RM_ (red) and CD8+ TIL (blue). Expression is depicted per treatment condition for *GAPDH* (household gene) and a series of cytotoxic T cell cytokines. Bars depict the median. (**B**) Overview of Ct-values determined using qPCR of CD39+CD103+ T_RM_ (red) and CD8+ TIL (blue) for the same conditions as depicted in A. qPCR for *IL13* was not determined (N.D.). Bars depict the median. (**C**) Correlation plots of Ct-values versus normalized counts of CD39+CD103+ T_RM_ (red) and CD8+ TIL (blue). The dashed line represents a reference correlation of 1 (x = y).

Activated CD39+CD103+ T_RM_ cells expressed several classical CD8-associated cytokine genes such as *IFNG* and *IL2*, plus several non-CD8 associated cytokines such as *IL21* and *CSF2*. Thus, we validated these findings by comparing log2-normalized expression between different treatment groups and Ct-values determined using qPCR for CD39+CD103+ T_RM_ and CD8+ bulk TIL (Figure 4A,B). Household gene *GAPDH* was included as an internal reference and control.

CD39+CD103+ T_RM_ and CD8+ TIL expressed some *IFNG* in resting condition, but minimal to no *TNF*, *IL2*, *IL10*, and *IL13*. CD39+CD103+ T_RM_ cells expressed low levels of *CSF2* (GM-CSF) in resting conditions, whereas CD8+ bulk cells did not (Figure 4A). Upon activation, both CD39+CD103+ T_RM_ and CD8+ TIL expressed *IFNG*, *TNF*, *IL2*, *IL10*, *IL13*, *IL21*, and *CSF2* mRNA (Figure 4A). The pattern in qPCR was very similar compared to mRNA expression. The qPCR for *IL13* was positive in water controls, therefore we have not included these and rendered them not determined. The normalized counts and Ct-values of *GAPDH* remained stable in different treatment settings in both populations, indicating the differences observed in cytokine genes are indeed a gene-specific effect of the PMA/ionomycin and actinomycin D treatments.

We observed a different response to actinomycin D in samples that were treated with both PMA and actinomycin. Despite pre-incubation with actinomycin, transcription of *IFNG*, *TNF*, and *IL2* was barely affected, whereas transcription of *IL10* and *IL13* strongly decreased. *IL21* and *CSF2* (GM-CSF) exhibited a somewhat intermediate profile, as combination of actinomycin + PMA lowered transcription, but did not halt it completely (Figure 4A,B). 

**Figure 5:**
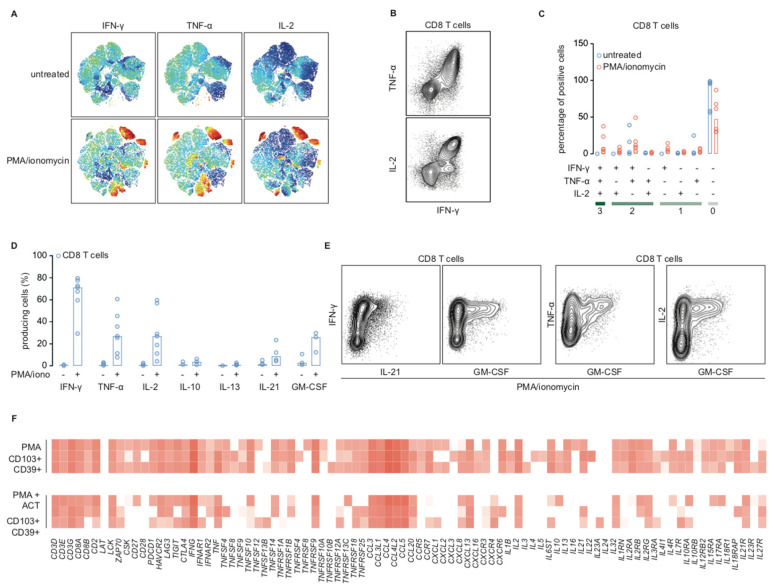
Activated CD8+ T cells are polyfunctional T cells that are responsive to (re)activation. (**A**) t-stochastic neighbor embedding plots of flow cytometry data of three endometrial tumor digests stained for CD3, CD8, IFN-γ, TNF-α, IL-2, and Zombie Aqua (live/dead stain). (**B**) Exemplary flow cytometry image of CD8+ T cells stained for CD3, CD8, IFN-γ, TNF-α, and IL-2. (**C**) Endometrial digests stained as described in (**B**) (*n* = 6). Percentages of untreated cells (blue) or cells activated by 4-h incubation with PMA/ionomycin (red) that produce no, one, two, or three of the depicted cytokines. Bars depict the median. (**D**) Endometrial cancer digests (*n* = 7) stained for CD3, CD8, IFN-γ, TNF-α, IL-2, and Zombie Aqua; 4 of these digests were additionally stained for IL-13 and IL-21, and 3 digests for IL-10 and GM-CSF. Percentages of untreated CD8+ TIL and CD8+ TIL activated by 4-h incubation with PMA/ionomycin positive for either of the depicted cytokines. Bars depict the median. (**E**) Exemplary flow cytometry images of CD8+ TIL stimulated for 4 h with PMA/ionomycin as depicted in D. (**F**) Heatmap of a customized set of T cell markers of library size-normalized, log2-transformed counts of PMA/ionomycin- and PMA/ionomycin + actinomycin D-treated CD39+CD103+ T_RM_ cells.

To validate our findings on the protein level, we first studied production of CD8+-associated cytokines IFN-γ, TNF-α, and IL-2 in endometrial cancer cytotoxic TIL. Endometrial cancer digests were incubated with a protein transport inhibitor containing Brefeldin A with or without PMA/ionomycin. t-Stochastic neighbor embedding (tSNE) of pooled data from three patient digests showed subsets of PMA-activated CD8+ TIL produced IFN-γ, TNF-α, and/or IL-2 (Figure 5A), whereas untreated cells did not (Figure 5A).

Of PMA-treated TIL, 31.8% (median of 6 samples) produced IFN-γ, TNF-α, IL-2, or a combination of these (Figure 5B,C), whereas only 3.8% of untreated cells produced any cytokine. In fact, 21.8% of activated cytokine-producing TIL were polyfunctional and expressed two or three of the studied cytokines (Figure 5C). Our sequencing and qPCR data indicated that T_RM_ and CD8+ TIL might have a more extensive cytokine repertoire that also includes IL-10, IL-13, IL-21, and GM-CSF. To validate this, we performed a flow cytometry experiment of PMA/ionomycin-activated and unstimulated CD8+ TIL from endometrial cancer digests including these markers plus IFN-γ, TNF-α, and IL-2 as an internal control. Activated CD8+ TIL in endometrial cancer barely produced IL-13 (1.06%), but they did produce GM-CSF (median 26.08%) and, to a lesser extent, IL-21 (median 8.79%) and very little IL-10 (median 3.7%) (Figure 5D). However, within unstimulated cells, only 2.75% produced GM-CSF, 1.475% produced IL-21, and 1.16% produced IL-10. Activated cells that produced either GM-CSF or IL-21 were polyfunctional, as they also produced IFN-y, TNF-α, and IL-2 (Figure 5E). This effect was specific to tumor-infiltrating lymphocytes, as CD8+ PBMCs did not produce cytokines after stimulation with PMA/ionomycin, even though they did express mRNA of several cytokines as determined by qPCR (Supplementary Figure S2A,B). Pretreatment with actinomycin D followed by PMA/ionomycin did result in transcriptional activation of several genes, albeit the rationale that actinomycin D halts all transcription. We observed a particular persistence of CCL chemokines, interleukin receptors and some, but not all, cytokines (Figure 4A,B and Figure 5F). Taken together, cytotoxic TIL are polyfunctional T cells with an extensive cytokine repertoire that includes production of IL-21 and GM-CSF.

### 2.5. T_RM_-Genes and PMA-Responsive Genes are Associated with Response to Immune Checkpoint Blockade in Publically Available Sequencing Data

Finally, we determined whether the T_RM_ and PMA-induced gene signature were associated with response to immune checkpoint inhibition. Therefore, we turned to publicly available sequencing data of melanoma samples that were taken prior to and upon treatment with immune checkpoint inhibitor nivolumab [30]. Our hypothesis was that PMA-responsive genes would be particularly seen in nivolumab-treated, i.e., reactivated, samples. Riaz et al. provided a list of differentially expressed genes of responders (complete response or partial response) versus non-responders upon treatment. Interestingly, several of our T_RM_-signature, stable, or PMA-responsive genes were indeed reported differentially expressed in responders during treatment, including *CD8*, *CD27*, *PDCD1*, *CTL4*, *LAG3*, *IFNG*, *PLEK*, *TNFRSF9*, *TNFRSF10A*, *CCL3*, and *CCL4* (Riaz et al. 2017, Supplementary Figure S3A–C) [30]. We also assessed expression of activation-responsive immune genes *TNF*, *IL21*, *IL10*, *IL13*, and *CFS2* in the ICB melanoma dataset, and found that *IFNG*, *TNF*, and interleukins *IL21* and *IL10* seemed to increase upon treatment in responders (Supplementary Figure S3C), even though they were not differentially expressed. However, *IL21R* was differentially expressed (DE) upon treatment irrespective of response, as were several IL2 receptor genes. Taken together, a number of genes associated with T_RM_ and PMA signatures were differentially expressed upon treatment with nivolumab in melanoma patients.

## 3. Discussion

We have analyzed baseline transcription and transcriptional stability in CD39+CD103+ T_RM_ in high-grade endometrial cancer and assessed transcriptional and translational responses upon T cell activation. In this study, we showed that resting CD39+CD103+ T_RM_ cells were transcriptionally active and expressed a characteristic tissue-resident transcriptional profile comparable to T_RM_ in other tumor types [31]. We observed expression of *ITGAE*, *ENTPD1*, *IFNG*, *TNFRSF9* (CD137), *TNFSF10, EGR2*, chemokines *CCL3*, *CCL4*, and *CCL5,* and chemokine receptors *CXCR3*, *CXCR4* and *CXCR6*. This is in line with the expression profile of CD103+ tumor-resident T cells in lung cancer, breast cancer, and ovarian cancer [9,18,19,26,32]. CD39+CD103+ T_RM_ cells are associated with an improved prognosis in many solid tumors [33] and CD103+ T_RM_ have been linked to response to immune checkpoint blockade [28,34]. This is further strengthened by the finding that the loss of E-cadherin, the receptor for the CD103/β7 integrin, inhibits responses to immune checkpoint blockade in melanoma [35]. In spite of the prognostic effect of CD103+(CD39+) T_RM_, their phenotype is often considered exhausted and functionally impaired across tumor types [9,19,26,36]. T_RM_ in our study indeed expressed several immune checkpoints including *HAVCR2, LAG3, CTLA4,* and *PDCD1*. Despite expression of these inhibitory receptors, CD39+CD103+ T_RM_ cells in this study upregulated markers of T cell activation, cytolytic activity, and cytokine production upon activation with PMA/ionomycin. In line with this, Abd Hamid et al. showed that activated T_RM_ cells express superior effector and resident memory profiles and have higher metabolic activity and mitochondrial capacity compared to non-activated controls [8]. Moreover, activated T cells in our study retained the functional capacity to produce cytokines TNF-α, IFN-γ, and IL-2. T cells that produce all three of these cytokines (triple-producers) are associated with superior activity upon rechallenge in viral infections, suggesting this may also hold true in tumor immunity. A subset of triple-producers also produced IL-21 and GM-CSF ex vivo, but not IL-13 or IL-10, even though we also observed mRNA expression of *IL10* and *IL13* in activated T_RM_. We do not fully comprehend why *IL21* and *CSF2* were translated, whereas *IL10* and *IL13* were not. We speculate that this could depend on environmental cues within the tumor, and therefore perhaps does not occur in vitro. Production of IL-21 by cytotoxic T cells is not extensively described, but *IL21* mRN*A* was found in PD1^high^ CD8+ T cells in a CAR-T solid tumor mouse model [37]. IL-21 promotes effector functions in naïve CD8+ T cells [38] and is important in CD8+ T cell survival, function, and memory formation [39]. Indeed, CD8+ T cells incubated with IL-21 showed enhanced tumor regression in an adoptive cell transfer mouse study [40]. Moreover, IL-21 stimulates B cell differentiation, memory B cell and plasma cell formation. We hypothesize that this may be an important mode of action for T_RM_-produced IL-21, as we have previously shown that CD103+ T_RM_ produce CXCL13 and associate with B cells and tertiary lymphoid structures in gynecological malignancies [18]. In line with this, Xiao et al. showed that IL-21 produced by CD8+ T cells promoted IgG production by matched B cells in vitro [41]. CD39+CD103+ T_RM_ also differentially expressed *CSF2* and activated CD8+ TIL produced GM-CSF ex vivo. GM-CSF, a potent immunostimulatory cytokine, is frequently used as an adjuvant in cancer vaccination trials and is linked to increased T cell recruitment to the tumor microenvironment [42].

We observed expression of *SPRY1, SPRY2, NR4A2,* and *NR4A3* in T_RM_ cells, and expression of these genes increased after T cell activation with PMA. Similarly, expression of *SPRY1* and *NR4A3* has been observed in CD103+ T_RM_ in lung cancer [19]. NR4A transcription factors and SPRY are initiated by NFAT (nuclear factor of activated T cells), and NFAT plays an important role in upregulation of CD103 on CD8+ T cells. *NR4A* knockdown in CD8+ CAR-T cells in a solid tumor mouse model increased CD8+ T cell effector functions and induced tumor regression [43]. Lack of Sprouty 1 and 2 enhances survival of effector CD8+ T cells [44], and mice with a T cell-selective deletion of Spry1 show enhanced responses to a tumor vaccine [45]. Reactivation of T cells as mimicked by PMA activation thus seems to initiate negative feedback mechanisms in T cells, suggesting that (re)activated T cells are functionally halted by endogenous negative feedback loops. In conclusion, CD39+CD103+ T_RM_ cells and CD8+ TIL in endometrial cancer are responsive to reactivation and produce both classical and non-classical cytokines that can influence both T cell and B cell function. Moreover, targeting activation-induced negative feedback mechanisms that halt T_RM_ activation, such as NR4A, could perhaps ameliorate responses to immune checkpoint blockade.

We have analyzed transcript stability in CD103+CD39+ T_RM_ by comparing untreated cells to cells treated with actinomycin D for 30 min followed by four hours of medium incubation prior to sequencing. Transcript stability prolongs the opportunity for translation, thereby increasing the bioavailability of the encoded protein. It is relevant to stabilize expression of (synthetic) mRNA in vitro and in vivo, as exploiting the unique properties of mRNA for therapeutic purposes gains more attention [46,47,48,49,50]. Von Niessen et al. have described how 3′ untranslated regions (UTRs) can augment the translation of synthetic mRNA in the context of vaccine antigens to elicit a T cell immune response [46]. As there are multiple genes whose 3′ UTR regulate mRNA stability, this could be a potential strategy to control therapeutic gene expression in viral vectors or vaccines. Mitochondrial transcripts do not possess a 3′ UTR, yet mitochondrial genes were among the most stable transcripts in our data. The stability of mitochondrial genes has not been elucidated fully but could perhaps be used in a similar manner as 3′ UTRs to stabilize desired mRNA.

We observed increased expression of several genes in actinomycin D-treated cells compared to controls, which could be the novo transcription via actinomycin D-insensitive routes. This included *USP36*, *ZNF528*, *MYO6*, and *EMC9*. One could hypothesize that the transcriptional halt provided by actinomycin does not affect all genes evenly, thereby allowing some genes to be less inhibited. It has been suggested that actinomycin D has preferential binding in CGG-triplet repeat sequences [51]. It could also be that these transcripts were not de novo described, but transcripts were relatively more abundant due to the decrease in the total number of transcripts after actinomycin D treatment and, therefore, they were found in this group. However, we have used a high concentration of actinomycin D that should be sufficient to inhibit all three eukaryotic polymerases [52], and a commonly used pre-incubation time [53]. Indeed, we observed almost complete inhibition of RNA synthesis in the actinomycin D monotherapy group, indicating effective inhibition of mRNA transcription.

Interestingly, several actinomycin-stable immune-related genes were particularly responsive to PMA activation. We hypothesized that the stability of these genes reflects a prolonged but dormant immune response, rendering these T_RM_ cells primed to rapidly respond once fully activated. Actinomycin D is a chemotherapeutic that is commonly used in several tumor types, particularly in sarcomas. In addition, it is sometimes used in in vitro studies to mimic the effect of platinum-based chemotherapy [54]. Platinum-based compounds act by binding to DNA and inhibiting its replication by forming cross-links in the DNA that block transcription, eventually resulting in cell death of dividing cells [55]. Actinomycin D inhibits RNA polymerase function by binding DNA at the transcription initiation complex, thereby preventing elongation of the RNA. Thus, effects of platinum-based compounds and actinomycin D on transcription in particular are presumed to be similar. This provides us with an opportunity to estimate TIL activation in the context of a read-out for adjuvant chemotherapy in patients with high-risk endometrial cancer. Remarkably, *IFNG*, *TNF,* and *IL2* were still increased (*IFNG*) or even appeared to be de novo transcribed (*TNF* and *IL2*) despite the inhibitory effect of actinomycin D on transcription compared to untreated samples. The hallmark cytokine profile of cytotoxic CD39+ T_RM_ cells does not seem to be affected by actinomycin D, suggesting that T_RM_ cells in vivo may preserve cytokine-producing capacities during chemotherapy. Some studies suggest platinum-based chemotherapy not only induces apoptosis via DNA damage but may also have immunogenic effects [56]. In addition, our group has recently studied the immune composition prior and after neoadjuvant platinum-based chemotherapy and found that the number of tumor-infiltrating lymphocytes in ovarian cancer was not affected by chemotherapy, although MHC-I expression was lower [57]. This is in line with findings by others who found CD8+ TIL infiltration to remain stable or even increase after neoadjuvant platinum-based chemotherapy in ovarian cancer [58,59,60,61], implying that platinum-based chemotherapy may augment a pre-existing immune response. As endometrial cancer, particularly MSI and POLE-mutant, is generally more immunogenic than ovarian cancer, this holds promise for patients with (advanced) endometrial cancer. In our study characteristic markers of T_RM_ cells in EC were responsive to PMA activation and were relatively unaffected by chemotherapy simulation, implying that a combination strategy of chemotherapy followed by immunotherapy may be an interesting option for further studies.

We have found that many of the transcriptional processes upon activation were not specific to CD39+CD103+ T_RM_ but seemed somewhat inherent to CD8+ TIL as a whole population. Upon examination of the data, we did observe large similarities in the response pattern between T_RM_ and CD8+ TIL. We have specifically made use of a non-otherwise specified CD8+ TIL population, as we were interested to study whether observed effects were also visible in a general population as you find it in a tumor. In line with previous experiments, the fraction of CD39+CD103+ TIL varied per patient, and therefore provided a representative image of the well-known variation in immune infiltrate in human tumors. As mentioned previously, high-grade endometrial cancer is a heterogeneous group of tumors. In this study, we performed sequencing analyses on two high-grade dMMR/MSI endometrioid tumors and one high-grade p53-mutant pMMR serous tumor. We did not observe general differences in transcription, and both PMA and actinomycin D responses were consistent between dMMR/MSI and MSS cases of high-grade EC, although we stress the small size of our group. This suggests that our findings are of interest to all high-grade EC.

Taken together, our findings showed that CD39+CD103+ T_RM_ cells in high-grade endometrial cancer are polyfunctional T cells with a reactivation-responsive repertoire, despite their exhausted phenotype, and these T_RM_ express a variety of immune checkpoints. Secondly, CD39+CD103+ T_RM_ showed increased transcript stability of PMA-responsive and mitochondrial genes, which may potentiate rapid responses upon T_RM_ cell reactivation in tumors. Thirdly, T_RM_ cells seem to incorporate activation-induced negative feedback mechanisms that halt T_RM_ activation, and targeting these may be of interest in optimizing response to immune checkpoint blockade.

## 4. Materials and Methods

### 4.1. Tumor Material Preparation

Endometrial cancer digests were obtained from surgical waste material collected in the University Medical Center Groningen, The Netherlands. Patients had given consent to use surgical waste material for research purposes. Samples were processed anonymously and assigned a coding number prior to storage. According to Dutch law no approval from our institutional review board was needed. Of the material used for sequencing, two tumors were high-grade endometrioid adenocarcinomas with MMR deficiency (FIGO IA, loss of PMS2, and FIGO IB, loss of MSH2 and MSH6, respectively) and the third tumor was of high-grade serous p53-mutant adenocarcinoma histology with proficient mismatch repair proteins, FIGO IIIC2. Tumors were cut into approximately 1cm^3^, enzymatically digested in RPMI medium (Gibco, Paisley, UK) with 1 mg/µL collagenase type IV (Gibco Life Technologies, Grand Island, NY, USA) and 12.6 µg/mL recombinant human DNase (Pulmozyme, Roche, Woerden, the Netherlands) for 30 min at 37 °C or overnight at room temperature. Digests were filtered using 70 µm cell strainers (Falcon) and enriched for peripheral blood mononuclear cells (PBMCs) using Ficoll-Paque PLUS (GE Healthcare Life Sciences, Marlborough, MA, USA). Cells were cryopreserved in fetal calf serum (FCS) with 10% dimethylsulfoxide and stored in liquid nitrogen until further use.

### 4.2. Isolation of PBMCs from Healthy Volunteers

Human PBMCs were isolated via Ficoll-Paque density gradient centrifugation (Ficoll-Paque PLUS, GE Healthcare Life Sciences, Marlborough, MA, USA) from buffy coats of healthy volunteers. Buffy coats were purchased from Sanquin and all donors gave informed consent (Sanquin Blood Supply, Groningen, the Netherlands). Isolated PBMCs were cryopreserved in FCS with 10% dimethylsulfoxide until use.

### 4.3. CD39+CD103+CD8+ and CD8+ T Cell Sorting

Endometrial cancer tumor digests from three endometrial cancer patients were thawed in RPMI + 10% fetal calf serum. Each digest was split four ways; one part remained untreated, one part was incubated with phorbol myristate acetate (PMA)/ionomycin (1× Cell Stimulation Cocktail, consist of 81 nM PMA and 1.34 µM Ionomycin, eBioscience, 00-4970-93, Waltham, MA, USA) for four hours, one part was treated with actinomycin D for 4.5 h (5 µg/mL, A1410-2mg, Sigma-Aldrich), and one part was pre-treated with 30 min of actinomycin D followed by four hours of PMA/ionomycin. All conditions were incubated at 37 °C. Next, samples were washed with PBS 2% FCS and stained with CD103-FITC, CD39-APC, CD3-PE, and CD8a-BV421 for 30 min at 4 °C (Supplementary Table S6). Cells were washed and filtered using a 35 µm strainer (Falcon). Propidium iodide (1 µg/mL) was used to exclude dead cells. Per treatment condition, 100 live CD3+CD8+ or 100 live CD3+CD8+CD39+CD103+ cells were sorted from all digests. Cells were sorted on a Beckman Coulter MoFlo Astrios sorter.

### 4.4. mRNA Sequencing CD39+CD103+CD8+ and CD8+ Tumor-Infiltrating Lymphocytes

Samples were sorted in 2 µL lysis buffer (0.2% Triton X-100 (Sigma-Aldrich) and 2U RNase inhibitor (Takara)) with 1 µL 10 µM oligo-dT primer and 1 µL 10 mM dNTP mix (Thermo Scientific). After sorting, the plate was spun down and incubated at 72 °C for 3 min. We used a modified SMARTseq2 protocol using custom-made primers (Supplementary Table S7), as described previously [18]. In brief, SmartScribe reverse transcriptase (Westburg-Clontech) and a template switching oligo (BC-TSO) were used to generate cDNA. Next, a PCR preamplification step was done with KAPA HiFi HotStart Ready Mix (Roche Diagnostics) and a custom-made PCR primer. The cDNA samples were purified using Ampure XP beads (Beckman Coulter) in a ratio of 0.6:1 (Ampure beads: cDNA). Samples were analyzed on a 2100 Bioanalyzer using a PerkinElmer LabChip GX high-sensitivity DNA chip (Agilent) and on a Qubit™ 4 Fluorometer (ThermoFisher Scientific) according to the manufacturers’ instructions. 500 pg of each sample were tagmented and N7xx and S5xx index adapters were used for barcoding according to the Illumina Nextera XT DNA sample preparation kit (Illumina). Thereafter, samples were purified with Ampure XP beads (ratio 0.6 Ampure: 1 cDNA) and analyzed on a 2100 Bioanalyzer. Samples were equimolar pooled (4 nM) and samples were sequenced on an Illumina Nextseq500 2500 using 75 bp single-end reads. The obtained mRNA sequencing data was demultiplexed into individual FASTQ files followed by alignment to the human reference genome hg38 using STAR (version 2.5.2).

### 4.5. qPCR of mRNA Sequencing Samples and Peripheral Blood CD8+ T Cells

Primers for GAPDH, IL-21, IL-13, IL-10, GMSC-F, TNFα, IFNγ, and IL-2 were ordered from Invitrogen Europrim (Supplementary Table S8). We performed qPCR in 384 well plates on a Bio-rad CFX384 Real-Time System. For each qPCR, a 0.5 µL cDNA sample (the same purified cDNA used for tagmentation) was used. Each reaction consisted of a final concentration of 1× PCR buffer without Magnesium (Invitrogen), 2.5 mM MgCl2 (Invitrogen), 0.2 mM dNTP mix (Invitrogen), 0.5 U Taq DNA polymerase (Invitrogen), 0.1 µM of each primer, 1x Evagreen dye (Biotium), and 0.5 µL cDNA.

### 4.6. Flow Cytometry

Endometrial cancer digests (*n* = 12) or frozen peripheral blood mononuclear cells were thawed, washed, and centrifuged. Pellets were resuspended in RPMI with 10% FCS. Half of each sample was incubated with PMA/ionomycin (eBioscience 1× Cell Stimulation Cocktail) for 4 h at room temperature, and all samples were incubated with a protein transport inhibitor to enable intracellular cytokine staining (1:1000, BD GolgiPlug™ Protein Transport Inhibitor, Thermo Fisher Scientific, BD555029, Waltham, MA, USA). Cells were stained with Zombie Aqua (Zombie Aqua™ Fixable Viability Kit, #423101, BioLegend) for 15 min, fixed, and permeabilized according to the manufacturer’s protocol (FIX & PERM™ Cell Permeabilization Kit, #GAS003, ThermoFisher Scientific), followed by incubation with antibodies for CD3, CD8α, TNF-α, IFN-γ, IL-2 with either IL-21 and IL-13 or IL-10, and GM-CSF (Supplementary Table S6). Samples were incubated at room temperature for 15 min, washed and suspended in PBS + 2% FCS. Samples were analyzed on FacsVerse (BD Biosciences) and results were analyzed with Cytobank premium software (cytobank.org).

### 4.7. Publically Available Sequencing Data

Fragments per kilobase million (FPKM) values and clinical data of melanoma samples prior and during Nivolumab treatment, as previously published by Riaz et al., were downloaded from the Gene Expression Omnibus (GEO) access number GSE91061 [30].

### 4.8. Statistics

Data were analyzed in R (R software, version 3.6.1) and GraphPad Prism (GraphPad Software Inc.). Differential expression was determined with DESeq2 package (1.26.0) in R, with a Benjamini–Hochberg correction. Heatmaps were visualized with the pheatmap package (1.0.12) in R. Guilt-by-association analysis was performed in Genet-ICA Network (manuscript in preparation) with default parameters and Biocarta pathways (accessible via http://www.genetica-network.com) [29]. Pathways with a z-score of >2 were considered relevant. Heatmaps of melanoma data were made with ComplexHeatmap package (2.2.0) and boxplots were made with ggplot2 package (3.3.0) in R. Throughout the work, significance was determined as (adjusted) *p*-value <0.05.

## Supplementary Materials

Supplementary materials can be found at https://www.mdpi.com/1422-0067/21/11/3770/s1.

## Abbreviations

ACT	Actinomycin D
CD	Cluster of Differentiation
EC	Endometrial cancer
FCS	Fetal calf serum
FIGO	International Federation of Gynecology and Obstetrics
GM-CSF	Granulocyte Macrophage – Colony Stimulating Factor
ICB	Immune checkpoint blockade
IL	Interleukin
MMR	Mismatch repair
- dMMR	Mismatch repair deficient
- pMMR	Mismatch repair proficient
MSI	Microsatellite instability
MSS	Microsatellite stability
ND	Not determined
PBMC	Peripheral blood mononuclear cells
PD	Progressive disease
PRCR	Partial response/complete response
PMA	Phorbol myristate acetate
POLE	Polymerase epsilon
SD	Stable disease
TIL	Tumor-infiltrating lymphocytes
TRM	Tissue-resident memory cell
tSNE	t-Stochastic neighbor embedding
UNK	Unkown
UTR	Untranslated region
