# Supplementary material for: Transcriptional Activity and Stability of CD39+CD103+CD8+ T Cells in Human High-Grade Endometrial Cancer
Source: Int J Mol Sci. 2020 May 27;21(11):3770. doi: 10.3390/ijms21113770 (PMC7312498; doi:10.3390/ijms21113770)
Supplement: Supplementary file 1 [file ijms-21-03770-s001.zip › ijms-803815 Supplementary/ijms-803815 Supplementary Figures.docx]

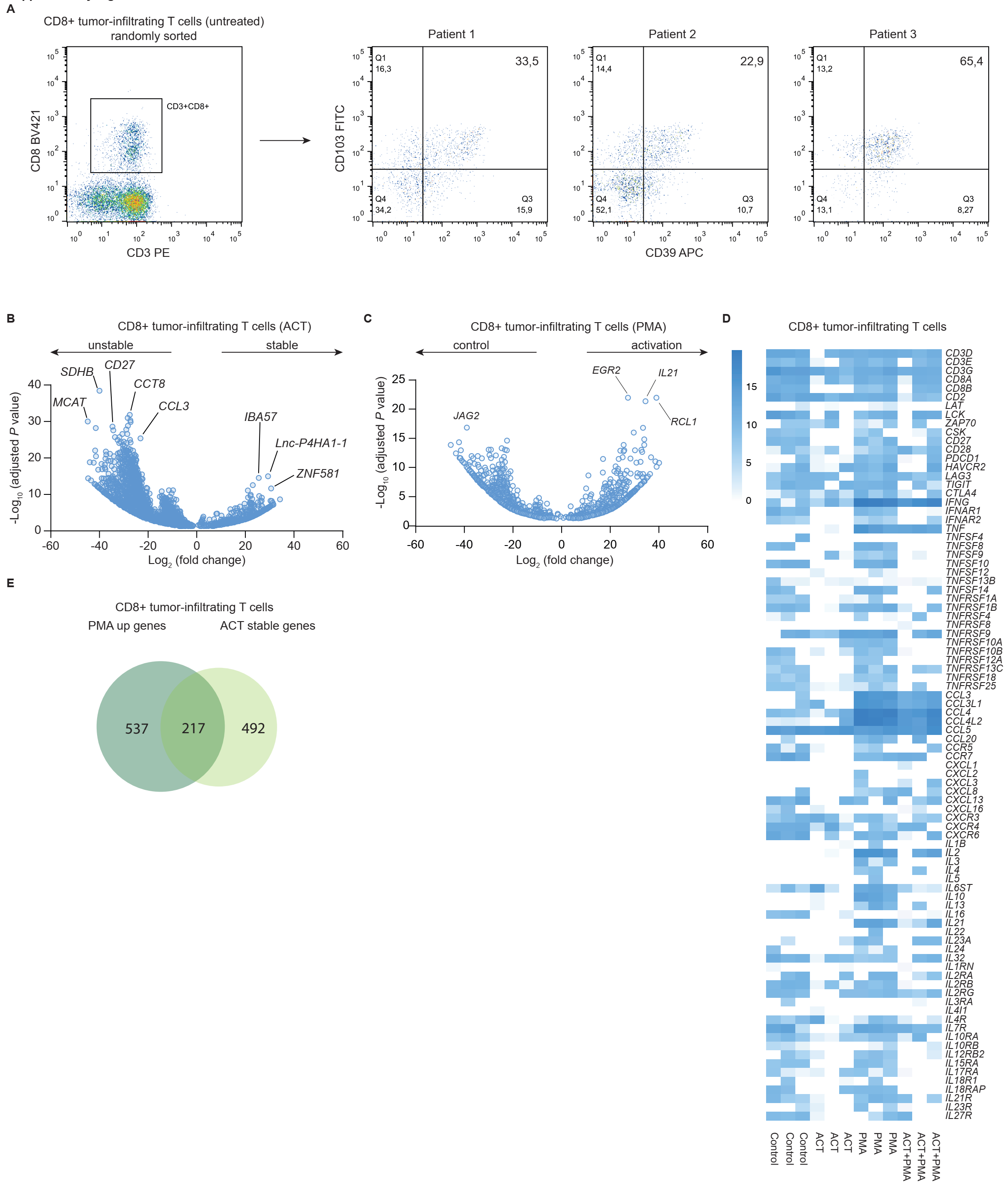


**Supplementary Figure S1.** Transcriptional profile of CD8+ bulk TIL upon transcriptional inhibition and activation in high-grade endometrial cancer. (**A**) Exemplary gating strategy of CD3+CD8+ TIL, CD8+ T cells were sorted randomly. On the right, the CD103 and CD39 expression within the CD3+CD8+ population per patient are depicted. (**B**) Volcano plot depicting differentially expressed genes (DEseq2) of CD8+ TIL treated with Actinomycin D for 4,5 hours versus untreated. Each dot represents a gene with a Benjamini-Hochberg adjusted p-value <0.05. Differentially decreased genes are depicted as unstable, differentially increased genes as stable. (**C**) Volcano plot depicting differentially expressed genes (DEseq2) of CD8+ TIL treated with PMA/ionomycin for four hours versus untreated. Each dot represents a gene with a Benjamini-Hochberg adjusted p-value <0.05. (**D**) Heatmap of a customized set of T cell markers of librarysize-normalized, Log2-transformed counts of CD8+ TIL, organized per treatment: untreated, 4.5 hours of Actinomycin D, 4 hours PMA/ionomyin or 30 minutes pre-incubation with Actinomycin D followed by 4 hours PMA/ionomycin. (**E**) Venn diagram showing overlap of differentially expressed genes (Benjamini-Hochberg-adjusted p<0.05) of CD8+ TIL after PMA versus significantly stable genes after Actinomycin D.


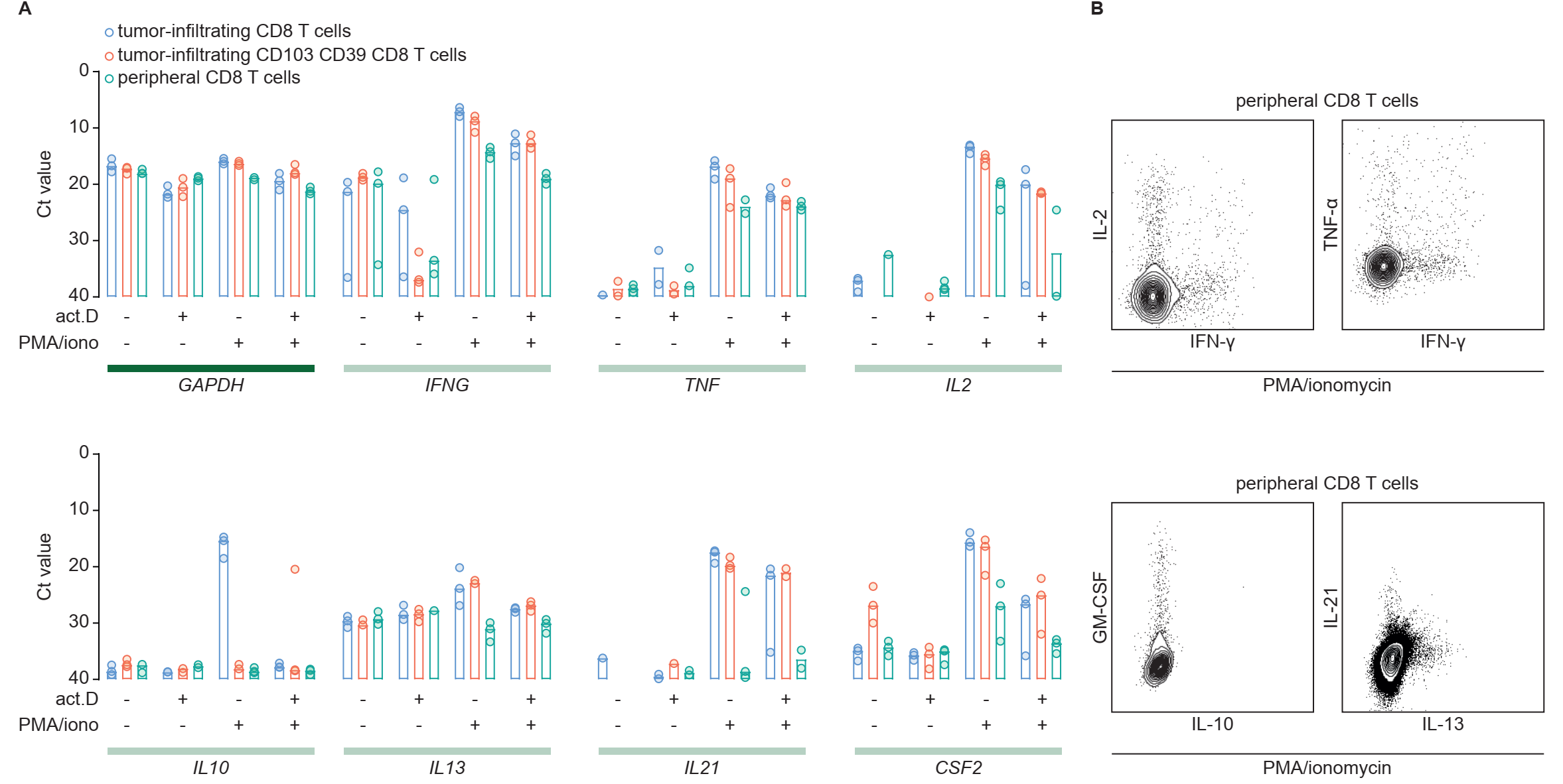


**Supplementary Figure S2.** CD8+ peripheral blood mononuclear cells do not produce IL-21 or GM-CSF upon stimulation with PMA/ionomycin. (**A**) Ct-values of cytokine genes determined by qPCR of CD8+ peripheral blood mononuclear cells (PMBCs, n=3) incubated for four hours with or without PMA/ionomycin with or without Actinomycin D as performed previously for TIL. (**B**) Flow cytometry image of CD8+ PBMCs stimulated for 4 hours with PMA/ionomycin depicting IFN-γ, TNF-α, IL-2, IL-10, IL-13, IL-21 and GM-CSF.


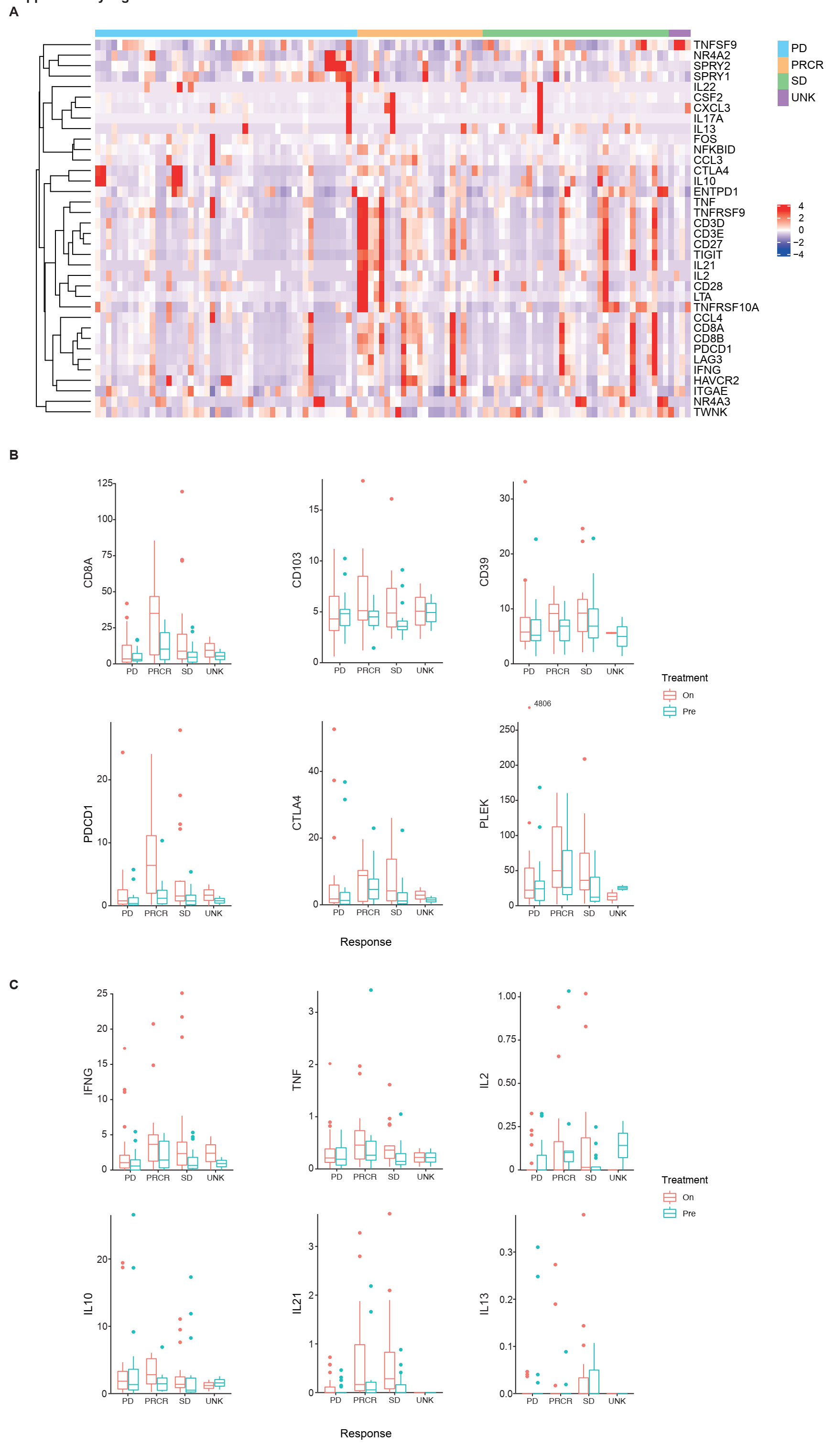


**Supplementary Figure S3.** T_RM_-genes and PMA-responsive genes are associated with response to immune checkpoint blockade in publically available sequencing data of melanoma (**A**) Heatmap of fragments per kilobase million (FPKM)-values of T_RM_ and PMA-responsive / stable genes in publically available sequencing data of melanoma samples pre- and on nivolumab treatment. Response is classified as progressive disease (PD), partial or complete response (PRCR), stable disease (SD) or unknown (UNK). (**B**) Boxplots of a subset of T_RM_ genes of FPKM-values as described in (A), comparing expression pre- and on treatment with nivolumab. (**C**) Boxplots of a subset of PMA-responsive cytokine genes of FPKM-values as described in (A), comparing expression pre- and on treatment with nivolumab.
